# Matrix Metalloproteinase 14 Mediates APP Proteolysis and Lysosomal Alterations Induced by Oxidative Stress in Human Neuronal Cells

**DOI:** 10.1155/2020/5917187

**Published:** 2020-11-16

**Authors:** Patricia Llorente, Soraia Martins, Isabel Sastre, Jesús Aldudo, María Recuero, James Adjaye, Maria J. Bullido

**Affiliations:** ^1^Centro de Biologia Molecular “Severo Ochoa” (C.S.I.C.-U.A.M.), Universidad Autonoma de Madrid, C/Nicolas Cabrera 1, 28049 Madrid, Spain; ^2^Centro de Investigacion Biomédica en Red sobre Enfermedades Neurodegenerativas, (CIBERNED), Madrid, Spain; ^3^Institute for Stem Cell Research and Regenerative Medicine, Medical Faculty, Heinrich-Heine-University Düsseldorf, Moorenstr. 5, 40225 Düsseldorf, Germany; ^4^Instituto de Investigación Sanitaria “Hospital la Paz” (IdIPaz), Madrid, Spain

## Abstract

The alteration of amyloid precursor protein (APP) proteolysis is a hallmark of Alzheimer's disease (AD). Recent studies have described noncanonical pathways of APP processing that seem partly executed by lysosomal enzymes. Our laboratory's *in vitro* human SK-N-MC model has shown that oxidative stress (OS) alters the lysosomal degradation pathway and the processing/metabolism of APP. The present study identifies the lysosomal protein matrix metalloproteinase 14 (MMP14) as a protease involved in the APP noncanonical processing. Previous expression analyses of the above cells showed MMP14 to be overexpressed under OS. In the present work, its role in changes in OS-induced APP proteolysis and lysosomal load was examined. The results show that MMP14 mediates the accumulation of an ≈85 kDa N-terminal APP fragment and increases the lysosome load induced by OS. These results were validated in neurons and neural progenitor cells generated from the induced pluripotent stem cells of patients with sporadic AD, reinforcing the idea that MMP14 may offer a therapeutic target in this disease.

## 1. Introduction

Alzheimer's disease (AD) is characterized by massive neuronal damage leading to cerebral atrophy and the loss of cognitive function. Sporadic AD, which accounts for more than 95% of all cases, is a highly complex disease for which neither the causal agents nor the molecular mechanisms involved are well known. It is widely accepted, however, that oxidative stress (OS), which is intimately linked to aging, is crucial in the onset and development of the disease. Using an *in vitro* free radical-generating system to simulate the OS associated with sporadic AD, our group has shown that such stress modulates the metabolism and proteolysis of APP [1] and that the lysosome axis is part of the mechanism linking OS with neurodegeneration, APP metabolism, and amyloidogenesis [2, 3].

APP processing is central to the pathophysiological mechanism of AD [4]. It is therefore of great interest to identify the proteases that might account for the APP proteolytic pattern seen in neurodegeneration models—not least because they might offer therapeutic targets. APP proteolysis, which is intimately linked with its intracellular trafficking, generates A*β* peptides plus multiple fragments with either neuroprotective or neurotoxic capacity [5]. Recent studies have revealed the proteolytic processing of APP to be more complex than just the canonical amyloidogenic and nonamyloidogenic pathways, with routes involving new secretases and their corresponding APP proteolytic fragments. The latter may accumulate in the brain of patients with AD, contributing to the synaptic dysfunction observed in the disease [6–8]. Remarkably, many of these noncanonical proteases act in the endolysosomal system, where endogenous APP seems to be mostly processed [9], highlighting the importance of the relationships between APP proteolysis and alterations in the above system.

In previous work, whole transcriptome analysis of SK-N-MC cells under OS induced by the xanthine-xanthine oxidase (X-XOD) system [2, 10] revealed the upregulation of MMP14 (also known as MT1-MMP and a member of the matrix metalloproteinase [MMP] family). This enzyme might therefore be part of the mechanism mediating the alterations in APP proteolysis induced by OS.

The MMPs belong to the metzincin group of proteases, which share a conserved zinc-binding motif in their active site. MMPs have been classically divided into six groups according to their known substrates in the extracellular matrix: collagenases, gelatinases, stromelysins, matrilysins, membrane-type MMPs, and other nonclassified MMPs [11]. More recent data illustrate an extremely wide range of substrates and interconnections for these enzymes, locating them at the crossroads of many biological processes [12, 13]. Soluble and membrane-bound MMPs are attracting increasing interest in neurodegenerative disease research, particularly AD, since they can cleave APP and A*β in vitro* and *in vivo* [14, 15].

MMP14 is phylogenetically close to MMP24, the APP *η*-secretase [7], with which it shows substrate cross-recognition [16]. These two MMPs render the same APP fragments [17]. Moreover, MMP14 directly cleaves APP [18] and promotes amyloidogenesis in human HEK293 cells overexpressing APPSwe. It also promotes the localization of APP/A*β* in endosomes, where A*β* production mainly occurs [19].

MMP14 is highly expressed in brain regions that show amyloid pathology and neuroinflammation. The upregulation of the active form of MMP14 has also been described in the hippocampus in 5xFAD model mice, and it is strongly associated with the accumulation of the *β*-secretase-derived fragment (*β*-CTF) and A*β* trimers [19]. When amyloid deposition and neuroinflammation occur, as in AD, reactive astrocytes and vascular smooth muscle cells markedly increase their expression of MMP14 which may then play a significant role in degrading soluble and deposited A*β* peptides [20]. Further, the abolishment of basal A*β* production by BACE-1 inhibition is rescued by MMP14, indicating that the latter might also mimic *β*-secretase-like activity [18]. APP proteolysis is, however, also heavily dependent on factors such as the cell type and its own level of overexpression [9].

The present work examines the role of MMP14 in the proteolysis of APP in a human cell line of neuronal lineage expressing basal levels of wild-type APP and in human neuronal cells differentiated from induced pluripotent stem cells (iPSCs) from patients with AD. These stem cells are among the most useful system available for the *in vitro* modelling of AD [21, 22]. Their use helps overcome the problem of the species gap between initial discoveries in rodent models and human studies and our inadequate understanding of the biology of AD [23]—which together may explain why so many clinical trials in AD have failed. Neither do studies with these cells suffer from the problem associated with postmortem brain samples, which, while vital for identifying the cellular and molecular changes associated with neurodegeneration, provide no chance to intervene in the course of disease [21]. In the present work, these cells reproduced the results obtained in SK-N-MC model with respect to MMP14 modulation by OS and regarding the participation of this enzyme in APP proteolysis and its effect on the lysosome load.

## 2. Materials and Methods

### 2.1. Cell Lines and iPSC-Derived Neurons

SK-N-MC cells (HTB-10), initially described as neuroblastoma and afterwards catalogued as part of the Ewing's sarcoma tumor family [24], were obtained from the American Type Culture Collection. These were cultured as a monolayer in minimal Eagle's medium supplemented with 10% heat-inactivated foetal calf serum, 1 mM sodium pyruvate, nonessential amino acids, 2 mM glutamine, and 50 *μ*g/mL gentamycin, at 37°C in a humidified 5% CO_2_ atmosphere.

#### 2.1.1. MMP14 Deficient Cells

The knockdown of MMP14 was performed by transfection of the above cells with a short hairpin RNA (shRNA) specific for MMP14 (Mission Control shRNA from Sigma, code TRCN0000050855); a scrambled shRNA (code SHC005) was used to generate the control cell line. Transfections were performed using jetPEI (Polyplus-transfection) according to the manufacturer's recommendations. At 48 h posttransfection, the cells were trypsinized and replated at low densities with the selection antibiotic puromycin. The clone line showing the lowest expression of MMP14 was selected for further studies. The culture conditions for these cells were the same as for the SK-N-MC parental line, with the selection antibiotic present throughout their growth.

#### 2.1.2. Induced Pluripotent Stem Cells

iPSCs were cultured in feeder-free culture conditions on Matrigel-coated (Corning) plates in mTeSR1 medium (StemCell Technologies). Cells were maintained at 37°C in a humidified 5% CO_2_ atmosphere and were split approximately every 5-6 days. These cells were manually groomed by removing any colonies with irregular borders, or that showed spontaneous differentiation or transparent centres prior to splitting.

#### 2.1.3. Neuronal Cells Derived from iPSCs

iPSC-derived neurons were cultured in a monolayer using a protocol based on a dual SMAD inhibition [25]. Briefly, to initiate differentiation, iPSCs were dissociated with TrypLE (Invitrogen), disaggregated to form a single-cell suspension, centrifuged at 100 x *g* for 5 min, and plated as a monolayer (day 0) at a concentration of 20,000 cells/cm^2^ in iPSC medium (mTeSR1). After reaching 90% confluence, the medium was changed to N2/B27 neural induction medium, i.e., DMEM/F12 with N2 (1%) and B27 (2%) supplements (Gibco), MEM-NEAA (Lonza) (1%), penicillin/streptomycin (Invitrogen), heparin (2 mg/mL, Sigma-Aldrich), fresh cAMP (Sigma, 1 *μ*M), and fresh IGF1 (Immuno Tools, 10 ng/mL), supplemented with dorsomorphin (200 ng/mL) and SB431542 (10 mM). The cells were split 11 days later using TrypLE and replated in neural differentiation medium, i.e., neurobasal medium with N2 and B27 supplements (Gibco), MEM-NEAA (Lonza) and penicillin/streptomycin (Invitrogen), fresh cAMP (Sigma, 1 *μ*M), BDNF, GDNF, and IGF-1 (all from ImmunoTools, 20 ng/mL), in 6-well plates coated with Matrigel® (Corning) to promote the adhesion and generation of neural progenitor cells (NPCs, an intermediate step along the differentiation path to neurons).

### 2.2. Cell Treatments

#### 2.2.1. Induction of Oxidative Stress

Exponentially growing cells at 80–90% confluence were placed in multiwell culture plates one day before treatment. Mild OS, that allowed the analysis of free radical-induced events preceding cell death, was induced as previously described [10] by adding xanthine (10 *μ*M) and xanthine oxidase (50 mU/mL) (X-XOD), obtained from Sigma and Roche, respectively. The effects were measured 24 h later.

#### 2.2.2. Pharmacological Inhibition of MMP14

The catalytic domains of MMPs show a high degree of structural homology which hinders the design of specific inhibitors for any particular enzyme. Modulators of their regulatory domains (e.g., the linker or hemopexin [PEX] domains), which affect their conformation, have the potential to be more specific in their action [26]. NSC405020 (APExBIO), a cell-permeable pentanylbenzamide compound that acts as an allosteric, reversible, and selective inhibitor of the collagenolytic activity of MMP14 [27], was added to the cells at a concentration of 100 *μ*M at the same time as X-XOD. The concentration of DMSO (vehicle) in the cell culture was 0.1% or lower and had no effect on any of the variables studied (not shown).

### 2.3. Cell Analysis

#### 2.3.1. Western Blotting

Western blotting (10% acrylamide resolving gels) was performed on cell extracts and different cell fractions as previously described [28]—except for CD63 for which nonreducing SDS-PAGE sample buffer was used—with the primary antibodies listed in Table 1 and horseradish peroxidase-coupled secondary antibodies. Immunodetection was performed using ECL™ Western Blotting Detection Reagents (GE Healthcare Life Sciences) according to the manufacturer's instructions. To quantify the intensity of the protein bands, densitometric analysis was performed using the Quantity One® Software (Bio-Rad).

#### 2.3.2. Immunofluorescence and Immunocytochemistry

Immunofluorescence assays were performed as previously described [1], employing the primary antibodies listed in Table 1 and Alexa Fluor-coupled secondary antibodies. Cells were examined using a LSM 710 laser scanning confocal microscope (Zeiss) coupled to a vertical M2 AxioImager (Zeiss) equipped with a 63X/1.4 Plan-Apochromat oil objective lens. Pictures were taken with a Spot RT digital camera (Diagnostic) using the Zeiss ZEN 2010 imaging system software. Images were processed using Adobe Photoshop CS3.

#### 2.3.3. Quantification of the Lysosome Load

The lysosome load was determined using the acidotropic probe LysoTracker Red DND-99 (LTR; Molecular Probes) via fluorogenic assays, as previously described [2]. The protein concentration of the lysates was quantified by the BCA method, and fluorescence was recorded using a FLUOstar® OPTIMA microplate reader (BMG LABTECH) (excitation wavelength 560 nm and emission wavelength 590 nm).

### 2.4. Statistical Analysis

Values in graphs are expressed as mean ± standarderrorofthemean (SEM). Differences among groups were analyzed using ANOVA. When the ANOVA analysis revealed a significant difference among the groups, pairwise comparisons were performed with the Student *t*-test. The SPSS Software v25 was used for the analyses. Significance was set at *p* < 0.05.

## 3. Results

### 3.1. Oxidative Stress Provokes the Accumulation of an APP N-Terminal Proteolytic Fragment (APP85)

**Figure 1:**
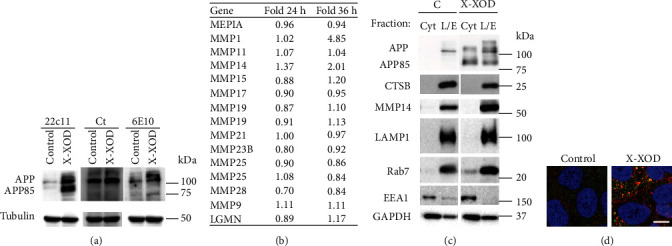
Modulation of APP proteolysis and MMP14 expression by oxidative stress. (a) SK-N-MC cells were treated with X-XOD for 24 h, and Western blotting then performed with anti-N-terminal APP (22C11), anti-C-terminal APP (Ct), anti-A*β* (6E10), and anti-*α*-tubulin antibodies (*n* = 3 independent experiments). (b) SK-N-MC cells were treated with X-XOD for 24 h or 36 h, and mRNA levels were analyzed in whole human genome expression microarrays as described in [2]. Candidate genes with basal expression levels above a given threshold (4.0 arbitrary units) were selected. Expression is shown as the fold change after X-XOD treatment compared to control cells at 24 and 36 h. In bold, *p* < 0.01. (c) SK-N-MC cells were treated with X-XOD for 24 h and then fractionated by differential centrifugation as described in [28]. Western blotting was performed using anti-APP (22C11), anti-MMP14, anti-LAMP1, anti-Rab7, anti-CTSB, anti-EEA1, and anti-GAPDH antibodies. The protein quantity determined by a BCA assay was used as a loading control (*n* = 3 independent experiments). Cyt: cytosolic-enriched fraction; L/LE: lysosome-late endosome-enriched fraction. (d) After incubation for 24 h, cells were examined by confocal microscopy. The representative panel shows immunofluorescence images for anti-N-terminal APP (green) and anti-MMP14 antibody (red). Original magnification: 63x. Scale bar: 10 *μ*m.

To analyze the effects of OS on APP processing, SK-N-MC human cells were treated with the free radical-generating system X-XOD [10]. Cells were analyzed after 24 h of treatment under conditions of minimal cell damage prior to apoptosis (Supplementary Figure 1). Western blot analysis of SK-N-MC total cell lysates with anti-APP antibodies—anti-N-terminal (22C11), anti-C-terminal, and anti-*β* amyloid (6E10)—revealed a prominent band with an approximate molecular weight of 85 kDa (Figure 1(a)) for the treated—but not the control—cells. This band corresponds to the N-terminal part of APP since it was recognized only by 22C11. In addition, sequencing by mass spectrometry of this band revealed it to contain N-terminal sequences of APP, but none of A*β* or CTF (not shown). According to the literature, this fragment is compatible with the action of noncanonical secretases, particularly MMP14, MMP24, and AEP [7, 8, 18]. It has been described that MMP14 and MMP24 cleave APP at the same site [17].

### 3.2. MMP14 Is Induced by OS and Is Mainly Localized in the Lysosome/Late Endosome Fraction

To select candidate noncanonical secretases able to account for the APP85 fragment, data available from previous whole genome expression microarray tests were used [2, 10] to examine the changes in mRNA levels induced by OS. MMP14 was the only one to be consistently upregulated in response to OS in all the models analyzed (Figure 1(b)).

Because APP is mostly located in the membranes of the endolysosomal system, where its processing mainly takes place [29], the subcellular location of APP and of the candidate protease (MMP14) was explored in SK-N-MC cells. As shown in Figure 1(c), under basal conditions (i.e., no treatment) both APP and MMP14 were preferentially located in the lysosome/late endosome-enriched fraction, together with the markers LAMP1, CTSB, and Rab7. In the X-XOD-treated cells, all the studied moieties, including APP, the APP85 fragment, MMP14, and the endolysosomal markers LAMP1 and Rab7, were clearly increased. Further, APP and MMP14 colocalized especially in large vesicles that accumulated in the treated cells (Figure 1(d)).

Altogether, these findings point to MMP14 as the best candidate responsible for the APP85 fragment induced by OS in SK-N-MC cells.

### 3.3. MMP14 Inhibition and Gene Silencing

**Figure 2:**
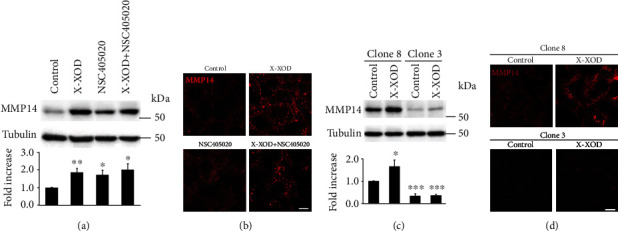
Modulation of MMP14 levels and the enzyme's subcellular location by oxidative stress. (a, b) SK-N-MC cells were treated with X-XOD in the presence or absence of NSC405020 for 24 h. (c, d) MMP14 deficient cells (clone 3) and control cells (clone 8), generated as described in Methods, were treated with X-XOD for 24 h. (a, c) Western blotting was performed using anti-MMP14 and anti-*α*-tubulin antibodies. The image shows a representative experiment, and the bar graph below the relative ratio versus control cells (mean + SEM) of the densitometric value of MMP14 normalized against *α*-tubulin. ^∗^*p* < 0.05 and ^∗∗^*p* < 0.01 (Student's *t*-test, *n* = 7 independent experiments for inhibited and *n* = 4 independent experiments for deficient cells). (b, d) After incubation for 24 h, cells were examined by confocal microscopy. The representative panel shows immunofluorescence images for anti-MMP14 antibody. Original magnification: 63x. Scale bar: 10 *μ*m. No staining was observed when the primary antibody was omitted.

To investigate the involvement of MMP14 in the changes in APP processing and the lysosomal pathway induced by OS in SK-N-MC cells, MMP14 was pharmacologically inhibited. To control for any potential side effects of the drug, stable gene silencing was also undertaken. MMP14 levels were checked by Western blotting using an antibody that recognizes the mature form (active enzyme).  Figure 2(a) shows that MMP14 increased significantly in SK-N-MC cells when treated with X-XOD. Immunofluorescence assays of these treated cells using the antibody specific for MMP14 revealed a different, more punctate location pattern in the presence of X-XOD (Figure 2(b)). A more condensed pattern was observed in the presence of the NSC405020 inhibitor, possibly due to its effect on the conformation of MMP14.

To confirm the efficient silencing of MMP14, Western blot analyses were performed on the deficient cells (Figure 2(c)), and a reduction in MMP14 levels of approximately 75% was observed. Moreover, no immunofluorescence signal for MMP14 was observed in these deficient cells (Figure 2(d)).

These data confirm the modulation of MMP14 by OS, as well as the validity of NSC405020 and the stable deficient cell line, as tools for examining the enzyme's role.

### 3.4. MMP14 Inhibition or Deficiency Prevents the Accumulation of APP85 Induced by OS

**Figure 3:**
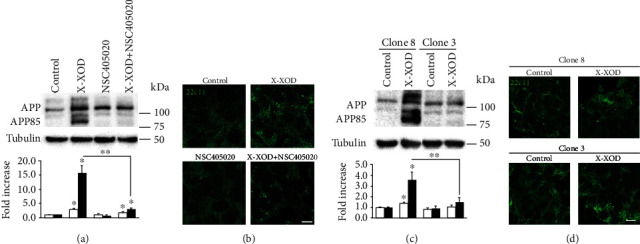
Effect of MMP14 inhibition or silencing on the accumulation of the APP 85 kDa fragment induced by OS. (a, b) SK-N-MC cells were treated with X-XOD in the presence or absence of NSC405020 for 24 h. (c, d) MMP14 deficient cells (clone 3) and control (clone 8) were treated with X-XOD for 24 h. (a, c) Western blotting was performed using 22C11 and anti-*α*-tubulin antibodies. The image shows a representative experiment, and the bar graph below the relative ratio versus control cells (mean + SEM) of the densitometric value of APP (white bars) and the APP85 fragment (black bars) normalized against *α*-tubulin. ^∗^*p* < 0.05 and ^∗∗^*p* < 0.01 (Student's *t*-test, *n* = 4 independent experiments). (b, d) After incubation for 24 h, cells were examined by confocal microscopy. The representative panel shows immunofluorescence images for 22C11 antibody. Original magnification: 63x. Scale bar: 10 *μ*m. No staining was observed when the primary antibodies were omitted.

The effect of the inhibitor NSC405020 on APP and APP85 levels was examined in cell lysates by Western blotting, using the antibody 22C11 (Figure 3(a)). The inhibitor produced a marked impairment in the increase in APP85 induced by X-XOD in the SK-N-MC cells. The same effect was evident in the deficient cells (Figure 3(c)).

In contrast, no significant effect of the inhibitor or gene silencing was seen on the levels of full-length APP induced by OS, indicating that the absence of the APP85 fragment is the result of APP proteolysis inhibition and not the consequence of reduced APP expression.

In the immunofluorescence assays with 22C11 antibody (Figure 3(b)), the cells showed increased signal and a different, more punctate pattern in the presence of X-XOD (Figure 3(b)). In the presence of X-XOD and the NSC405020 inhibitor, a reduction in immunofluorescence was seen with respect to X-XOD treatment alone; these results correlate with the Western blot result in which the APP band decreased, and the APP85 band even disappeared. In the deficient cells, immunofluorescence assays also showed an increase in APP levels and a different pattern in the presence of X-XOD (Figure 3(d)).

Taken together, these results support the involvement of MMP14 in the regulation of APP metabolism by mild OS. The inhibition or gene silencing of MMP14 almost completely prevented the accumulation of the APP85 band in response to OS, supporting a direct role of MMP14 in APP proteolysis, as has been described in other models [15].

### 3.5. MMP14 Inhibition or Deficiency Diminishes the Increase in the Lysosome Load Induced by OS

Increasing evidence indicates that the lysosomal system is altered in AD. Lysosomal hydrolase-containing compartments are massively accumulated in atrophic and degenerating neurons and their processes. These hydrolases are then released into the extracellular space where they then contribute to the formation of senile plaques [30]. In addition, the increased expression of lysosomal enzymes is observed during the early stages of AD [31], and the lysosomes of neuronal cells are rich in APP which is rapidly trafficked towards them [32]. Thus, the endolysosomal system may play a central role in the pathophysiology of AD and specifically in the type of proteolysis that APP undergoes [9, 33]. The potential role of MMP14 in the modulation of the lysosome system by OS was therefore explored in the SK-N-MC model by analyzing the effect of its inhibition and gene silencing on the lysosome load changes induced by OS.

**Figure 4:**
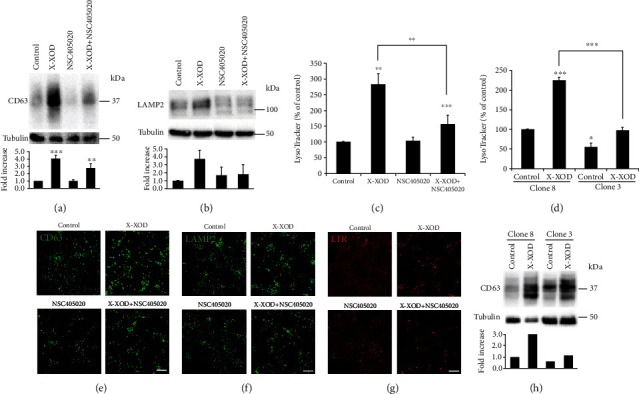
Effect of MMP14 inhibition or gene silencing on the increase in lysosome load induced by OS. (a–c, e–g) SK-N-MC cells were treated with X-XOD in the presence or absence of NSC405020 for 24 h. (d, h) MMP14 deficient cells (clone 3) and control (clone 8) were treated with X-XOD. (a, b) Western blotting was performed using (a) anti-CD63, (b) anti-LAMP2, and anti-*α*-tubulin antibodies. The image shows a representative experiment, and the bar graph below the relative ratio versus control cells (mean + SEM) of the densitometric value normalized against *α*-tubulin.^∗^*p* < 0.05 and ^∗∗^*p* < 0.01 (Student's *t*-test, *n* = 4 independent experiments). (c, d) After incubation for 24 h, the lysosome load was analyzed by fluorimetric measurement using LysoTracker. The graph shows the mean (±SEM) fluorescence expressed as a percentage of the control value. ^∗^*p* < 0.05, ^∗∗^*p* < 0.01, and ^∗∗∗^*p* < 0.01 (*t*-test, *n* = 4 independent experiments). (e–g) After incubation for 24 h, cells were examined by confocal microscopy. The representative panel shows immunofluorescence images for (e) anti-CD63, (f) anti-LAMP2, and (g) LysoTracker. Original magnification: 63x. Scale bar: 10 *μ*m. No staining was observed when the primary antibodies were omitted. (h) After incubation for 24 h, control or MMP14 deficient cells were examined by Western blotting using anti-CD63 and anti-*α*-tubulin antibodies.

The lysosomal markers CD63 and LAMP2 were measured by Western blotting (Figures 4(a) and 4(b), respectively). As expected, CD63 and LAMP2 increased in the cells treated with X-XOD. However, this increase was prevented in cells treated with NSC405020 and in the deficient cell line. In the deficient cells (Figure 4(h)), CD63 also decreased in comparison to nondeficient cells in the presence of OS.

The lysosome load was measured using the acidotropic probe LysoTracker. As shown in Figure 4(c), X-XOD-treated cells showed a significant increase in LysoTracker fluorescence, whereas the increase in cells treated with X-XOD and NSC405020 was significantly smaller (*p* < 0.01). In the deficient cells (Figure 4(d)), LysoTracker fluorescence decreased significantly in comparison to nondeficient cells, and this decrease was more pronounced in X-XOD-treated cells than under basal conditions (untreated cells). These results indicate that MMP14 deficiency impairs the increase in lysosomal markers induced by OS.

The intracellular location pattern of several endolysosomal markers was also studied using immunofluorescence assays. As observed in Figures 4(e)–4(g) (CD63, LAMP2 and LysoTracker, respectively), all the markers increased in the presence of OS, but this increase was impaired in cells treated with X-XOD in combination with NSC405020. These results correlate with those obtained by Western blotting and with the LysoTracker fluorimetric quantifications, indicating that MMP14 inhibition prevents the increase in the lysosome load induced by OS.

Taken together, these results suggest that MMP14 is part of the mechanism that mediates the connection between OS, lysosomal alterations, and APP proteolysis in the SK-N-MC cell model.

### 3.6. Differentiation of iPSCs into Neural Cells

APP proteolysis is highly dependent on cell type and species [9]. Cells derived from the iPSCs of patients with AD were therefore used to validate the results obtained with the SK-N-MC model. Two iPSC cell lines (here termed lines 126 and 105) were derived from the lymphoblasts of patients who were APOE4 homozygous and carriers of the risk variant p. R47H of TREM2. These lines have been previously characterized by Martins et al. [34, 35].

**Figure 5:**
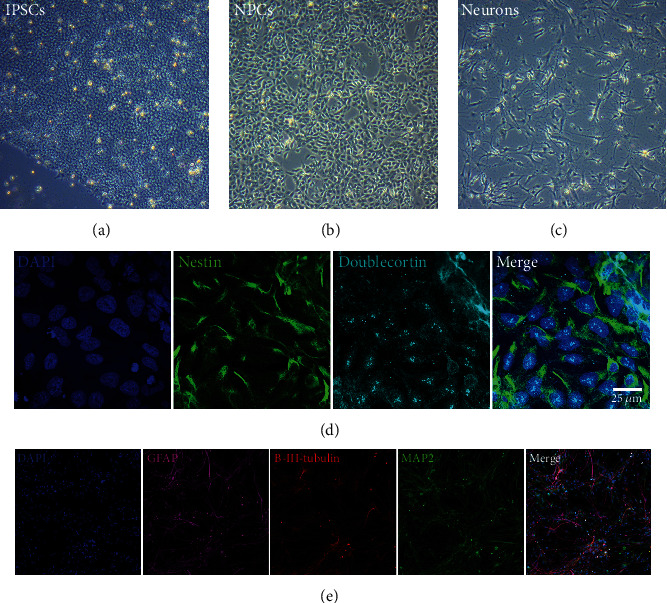
iPSC differentiation into neuronal cells following the dual SMAD inhibition protocol. (a) iPSCs, (b) NPCs, and (c) neurons. Images were taken under the phase contrast microscope. Original magnification: 10x. (d) Characterization of NPCs. Immunofluorescence assay positive for nestin and doublecortin. Nuclei stained with Hoechst. Original magnification: 63x. Scale bar: 25 *μ*m. (e) At advanced stages of differentiation, neurons were the major cell type. Immunofluorescence assay positive for MAP2, *β*-III tubulin, and GFAP—markers for neurons and astrocytes, respectively. Nuclei stained with Hoechst. Original magnification: 10x.

To obtain differentiated neural cells (Figure 5(a)), a dual SMAD inhibition protocol was followed [25]. NPCs were prepared from iPSC cell line 126 and fully fledged neurons from the iPSC cell line 105 (Figures 5(b) and 5(c), respectively).

The NPCs had the typical morphology of this cell type under the microscope (Figure 5(b)). Antibodies specific for nestin, a marker of ectoderm-differentiated cells, and for doublecortin confirmed their identity. Nestin, a neural stem cell marker, is required for the survival and proliferation of NPCs, while doublecortin is a microtubule-associated protein required for the normal migration of neurons into the cerebral cortex. As shown in Figure 5(d), the cells derived from iPSC 126 were positive for both nestin and doublecortin, confirming them to be NPCs.

The differentiation of iPSC line 105 led to a mix of cell types similar to that seen in the brain (Figure 5(c)). As shown in Figure 5(e), immunofluorescence tests for MAP2 (a neuron marker), GFAP (an astrocytes marker), and beta III tubulin (a marker of nerve cells, including neurons and astrocytes) identified these different cell types; the majority, however, were neurons.

### 3.7. Role of MMP14 in iPSC-Derived Human Neural Cells

Cells derived from iPSCs were subjected to OS in the same manner as were the SK-N-MC cells, both in the absence or presence of the MMP14 inhibitor, NSC405020.

**Figure 6:**
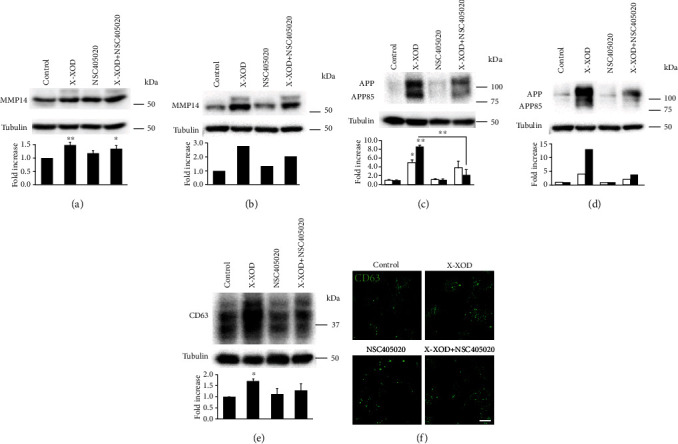
Validation of results in human iPSC-derived neural cells. (a, c, e, f) NPCs and (b, d) neurons were treated with X-XOD in the presence or absence of NSC405020 for 24 h. Western blotting was performed using (a, b) anti-MMP14, (c, d) 22C11, (e) anti-CD63, and anti-*α*-tubulin antibodies. The image shows a representative experiment, and the bar graph below the relative ratio versus control cells (mean + SEM) of the densitometric value (APP (white bars) and APP85 fragment (black bars)) normalized against *α*-tubulin. ^∗^*p* < 0.05 and ^∗∗^*p* < 0.01 (Student's *t*-test, NPCs *n* = 3 independent experiments and neurons *n* = 1; CD63 *n* = 2 independent experiments). (f) After incubation for 24 h, NPCs were examined by confocal microscopy. The representative panel shows immunofluorescence images for anti-CD63 antibody. Original magnification: 63x. Scale bar: 10 *μ*m.

Western blotting showed NPCs and neurons treated with X-XOD to have markedly higher MMP14 levels (Figures 6(a) and 6(b), respectively), similar to the effect observed in the SK-N-MC model (Figure 2). The effect of X-XOD appeared larger in the neurons. MMP14 was also analyzed by immunofluorescence assays in NPCs, and the same effect of X-XOD-treatment as in the SK-N-MC model was seen (Supplementary Figure 2).

The effects of mild OS and the inhibitor NSC405020 on APP processing were examined in cell lysates of NPCs and neurons by Western blotting using the antibody 22C11 (Figures 6(c) and 6(d), respectively). Similar to that seen with the SK-N-MC model, the main effect of OS was the induction of the APP85 band and its almost complete inhibition by NSC405020. Similar to what occurred in the SK-N-MC model, the relative APP85/APP quantity in cells subjected to OS was significantly lower when in the presence of the inhibitor. This indicates that MMP14 is involved in the proteolytic processing of APP resulting in the formation of APP85 in iPSC-derived NPCs and neurons.

To analyze the lysosome load, CD63 levels were measured in NPCs by Western blotting (Figure 6(e)), which returned similar results to those obtained in the SK-N-MC model (Figure 4). An increase in CD63 level was observed in X-XOD-treated NPCs that were impaired when NSC405020 was also present. NPCs were also subjected to immunofluorescence assays.  Figure 6(f) shows an increase in CD63 in the presence of X-XOD that was impaired in the presence of the MMP14 inhibitor. The same effect was observed when the fluorescence of the LysoTracker probe was analyzed (Supplementary Figure 2). These results agree with those obtained in the Western blotting assays and with those obtained with the SK-N-MC cells (Figure 4).

These results show that the inhibition of MMP14 in human iPSC-derived NPCs and neurons modulates the change in APP processing—more specifically the accumulation of the APP85 fragment—and in the lysosome load induced by OS.

## 4. Discussion

### 4.1. MMP14 Regulates Changes in APP Metabolism/Processing Induced by OS in SK-N-MC Cells

Numerous risk factors have been associated with the onset of AD, one of which is aging-associated OS [36, 37]. OS modulates APP metabolism and proteolytic processing. Since APP processing is central to AD pathogenesis, the mechanisms linking OS to APP proteolysis could offer therapeutic targets. Our group previously reported the involvement of OS in APP processing [1], the connection between APP proteolysis and the lysosome system in response to OS [3], and the role of the lysosomal protease CTSB in the accumulation of A*β* oligomers in response to OS [28].

Recently, the proteolytic processing of APP has been shown more complex than once thought, with new secretases and the fragments they produce having been identified [6, 15]. Also, there appears to be a strong correlation between APP proteolysis and its intracellular trafficking, with the vesicles of the endolysosomal system being the structures in which the amyloidogenic processing of APP mainly takes place [6, 9]. Further, it has been shown that OS alters APP metabolism and affects protein degradation pathways, including the lysosomal system [2, 38], suggesting a relationship exists between the proteases associated with the lysosomal system and the APP proteolysis alterations induced by OS. Within this context, our group has worked to identify the proteases responsible for the alterations in APP proteolysis induced by OS in SK-N-MC cell model. The present work focuses on the most prominent Western blot band induced by OS, i.e., APP85, an 85 kDa N-terminal fragment of APP—that was stained only by 22C11 anti-APP N-terminal antibodies. According to the literature, several potential noncanonical secretases are compatible with the mobility and sequence of this fragment, especially MMP24, AEP, and MMP14 [7, 8, 18]. Data collected in previous studies performed at our laboratory showed only mRNA for MMP14 to be significantly increased under OS conditions in SK-N-MC cells (Figure 1). MMP14 mRNA was also induced by OS in all the SK-N-MC cell models available in our group, i.e., cells infected by HSV-1 or overexpressing the APPwt or APPSwe variants (mean fold increase 2.31, *p* 0.001, unpublished results). These findings show MMP14 to be the strongest candidate producer of APP85. Indeed, in the present work, its inhibition or gene silencing caused significant reductions in APP85 (and even its disappearance) under OS conditions.

The ability of several MMPs to degrade APP and induce the aggregation of A*β* plus the evidence of increased expression of MMPs detected in postmortem brain tissue of AD patients indicate that MMPs play an important role in the pathogenesis of AD [39]. However, designing effective *in vivo* inhibitors for this class of enzymes is extremely challenging given the broad structural similarity of their active sites and the dynamic functional interconnectivity of MMPs with other proteases, their inhibitors, and substrates (the so-called degradome) in healthy and diseased tissues [40]. Moreover, no such inhibitor has been successfully developed as an anti-AD drug, mainly because of harmful side effects; the low specificity of MMP inhibitors is a real challenge in this respect. The dual action of MMPs complicates the use of broad-spectrum MMP inhibitors, and more research will be needed if we are to understand the diverse roles of these proteases and design drugs and therapeutic strategies specific for AD [39] and other neurodegenerative diseases based on MMP inhibition [15].

The mechanism of action of inhibitors specific for MMP14, such as NSC405020, may be directed through the allosteric modification that the PEX domain exerts on the catalytic domain [26]. This would directly prevent APP acting as a substrate for MMP14 and do so indirectly via the effect on other MMPs given the upstream position of MMP14 in the activation cascade.

Recent reports on the ability of MMP14 to directly cleave APP in mouse models and in an embryonic human HEK293 cell line overexpressing APPSwe show that part of the effect of this protease involves the direct proteolysis of APP to render an sAPP95 fragment [18], which probably corresponds to the APP85 reported here.

### 4.2. MMP14 Regulates the Increase in the Lysosome Load Induced by OS

The existence of a link between lysosome dysfunction and neurodegeneration has been reported by numerous research groups. It is now under intense investigation given its potential to offer a pharmacological target [41, 42]. Neurons are especially vulnerable to lysosome dysfunction and rely strongly on functional autophagic and endocytic pathways. Without them, these post mitotic cells are unable to dilute debris and undigested material. Abnormalities of the lysosomal system in AD include very early-appearing endosome enlargement, the accumulation of autophagic vesicles, increased lysosome biogenesis, and lysosomal proteolysis deficits [43]. Interestingly, it has been shown that activated or enlarged endosomes containing soluble A*β* appear prior to the deposition of amyloid plaques in AD and Down's syndrome [44].

These abnormalities have also been reported in AD models and in our laboratory's cell models of HSV-1 infection and OS [2, 45], highlighting the involvement of the lysosomal system in CNS pathologies and supporting the hypothesis that lysosomal dysfunction mediates the neurodegeneration induced by HSV-1 and OS.

A relation of MMP14 with lysosomal dysfunction has been reported by Mori et al. [46], who showed an increase in the number and size of endocytic and autophagic organelles in the mammary gland of MMP14 KO mice. In the present SK-N-MC model, we do not detect these changes under basal culture conditions, most probably due to differences between the models studied, including cell lineage and species. However, our data show that MMP14 participates in the alterations of lysosome load induced by OS in human neuronal cells, indicating the existence of functional connections of MMP14 with the lysosomal system in response to OS.

### 4.3. The Results Obtained with Neuronal Cells Differentiated from the iPSCs of Patients with AD Confirm the Involvement of MMP14 in APP Proteolysis and Lysosomal Alterations Induced by OS

IPSCs of human origin allow studies to be performed in a disease-relevant context. Although further work is needed to produce the full repertoire of cells in the mature brain [22], these cells can help speed the transition of ideas from bench to bedside [21]. In the present work, iPSC lines obtained from patients with AD expressing the TREM2 R47H variant [34, 35] were differentiated and used to validate the results obtained in the SK-N-MC cell model. The p. R47H variant of TREM2 is a risk factor for AD of comparable importance to that of APOE *ε*4 [47]. This variant impairs protein function, limiting the ability to clear amyloid and cellular debris, suggesting that the link between this gene and the disease lies in an imbalance in cellular protection mechanisms [48].

The present work showed both NPCs and fully differentiated neurons to reproduce the behavior of the SK-N-MC cells, the pattern of APP proteolysis, and the increase in the lysosome load, in response to OS. They also showed the same behavior with respect to the modulation of MMP14 by OS.

In summary, the present results show that MMP14 plays a role in the modulation of APP proteolysis induced by OS in human neurons, at least in part due to the direct proteolytic processing of APP. These findings are in line with previous reports showing the proamyloidogenic effects of this protease in mice and in the HEK cell line [18]. The observation that MMP14 is involved in the mechanism by which mild OS induces lysosomal alterations and changes in APP proteolysis in AD-patient-iPSC-derived neurons reaffirms this protease to offer a potential pharmacological target.

## 5. Conclusions

Oxidative stress (OS) induces the accumulation of an approximately 85 kDa N-terminal fragment of APP (APP85) and also produces an increment of lysosomal load. MMP14 mediates the APP85 accumulation and the increase in lysosomal load induced by OS. These effects mediated by MMP14 are also observed in NPCs and neurons differentiated from iPSCs of Alzheimer's disease patients. Our findings support the potential of MMP14 as an Alzheimer's disease pharmacological target.

## Figures and Tables

**Table 1:** Primary antibodies used in Western blotting and immunofluorescence assays.

Antigen	Host	Dilution		Reference
		WB	IF	
22C11	Mouse	1 : 4000	1 : 50	Millipore (MAB348)
6E10	Mouse	1 : 500	1 : 50	Covance (SIG-39300)
C-Terminal	Rabbit	1 : 1000	1 : 100	Sigma (A8717)
MMP14	Rabbit	1 : 2000	1 : 100	Abcam (ab51074)
CD63	Mouse	1 : 100	1 : 50	DSHB (H5C6)
LAMP1	Mouse	1 : 1000	---	DSHB (H4A3)
LAMP2	Mouse	1 : 1000	1 : 50	DSHB (H4B4)
Rab7	Rabbit	1 : 1000	1 : 100	Cell Signaling Technology (9367)
CTSB	Rabbit	1 : 500	---	Santa Cruz (sc-13985)
Tubulin	Mouse	1 : 10000	---	Sigma (T5168)
CD63	Mouse	1 : 100	1 : 50	DSHB (H5C6)
Nestin	Rabbit	---	1 : 200	Sigma Aldrich (N5413)
MAP2	Rabbit	---	1 : 200	SySy (188002)
GFAP	Guinea pig	---	1 : 200	SySy (173004)
Doublecortin	Guinea pig	---	1 : 200	Millipore (AB2253)
*β*-III-tubulin	Mouse	---	1 : 200	Cell Signaling Technology (4466S)

## Data Availability

Data are available on request. María J Bullido should be contacted to request the data.

## Supplementary Materials

Supplementary Materials Supplementary Figure 1: X-XOD system in SK-N-MC and effects prior to apoptosis. Supplementary Figure 2: NPC assays with MMP14 and LysoTracker show the same results than SK-N-MC cells.
