# Supplementary material for: Matrix Metalloproteinase 14 Mediates APP Proteolysis and Lysosomal Alterations Induced by Oxidative Stress in Human Neuronal Cells
Source: Oxid Med Cell Longev. 2020 Nov 16;2020:5917187. doi: 10.1155/2020/5917187 (PMC7685830; doi:10.1155/2020/5917187)
Supplement: Supplementary Materials — Supplementary Figure 1: X-XOD system in SK-N-MC and effects prior to apoptosis. Supplementary Figure 2: NPC assays with MMP14 and LysoTracker show the same results than SK-N-MC cells. [file 5917187.f1.docx]

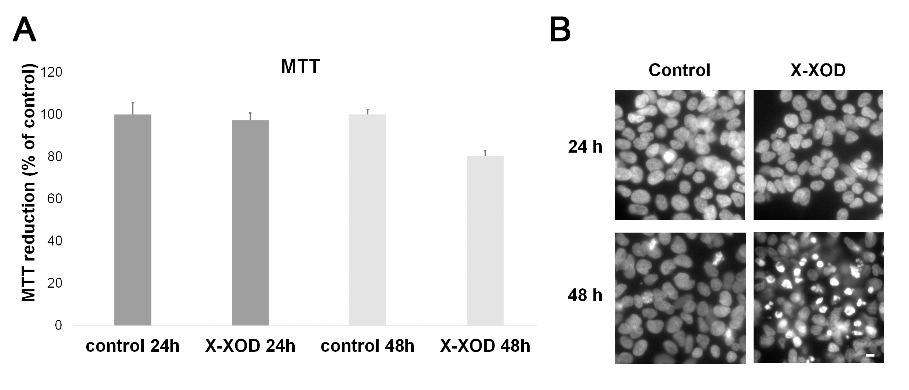


**Supplementary Figure 1. X-XOD system in SK-N-MC, effects prior to apoptosis.** Cells were treated with X-XOD for 24 or 48 h to confirm the conditions of minimal cell damage prior to apoptosis. A) After incubation for 24 or 48 h, the cell viability was analyzed by MTT assay. The graph shows the mean (±SEM) fluorescence expressed as a percentage of the control value. B) After incubation for 24 or 48 h, cells were examined by confocal microscopy. The representative panel shows immunofluorescence images for DAPI, with fragmented and condensed chromatin at 48 h. Scale bar: 10 µm.


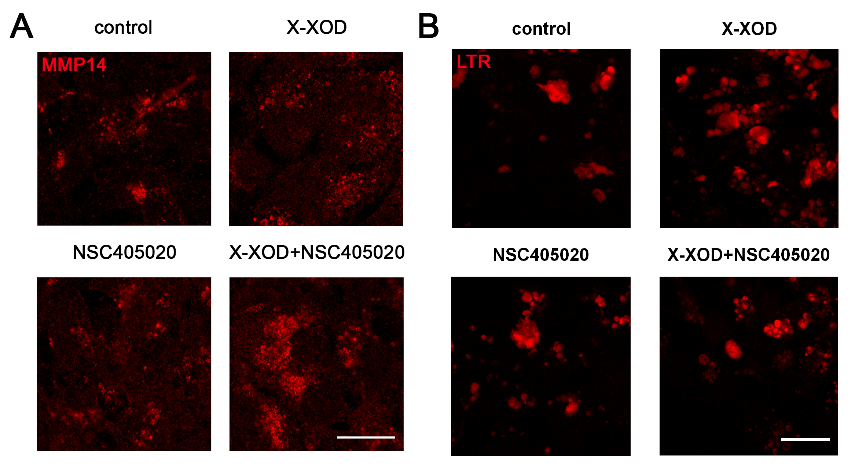


**Supplementary Figure 2. NPCs assays witn MMP-14 and LysoTracker show the same results than SK-N-MC cells.** NPCs were treated with X-XOD in the presence or absence of NSC405020 (100 μM). After incubation for 24 h, cells were examined by confocal microscopy. The representative panel shows immunofluorescence images for A) anti-MMP14 antibody and B) LysoTracker probe. Original magnification 63×. Scale bar 10 μm.
